# Genetic Variability and Conserved T-Cell Epitope Prediction of the HPV16 E1 Protein in Cervix Samples from Peru

**DOI:** 10.3390/pathogens15050526

**Published:** 2026-05-13

**Authors:** Eliezer Bonifacio-Velez de Villa, Miguel Angel Aguilar-Luis, Wilmer Silva-Caso, Lorena Becerra-Goicochea, Yordi Tarazona-Castro, Deysi Aguilar-Luis, Dayana Denegri-Hinostroza, Angela Cornejo-Tapia, Ronald Aquino-Ortega, Juana del Valle-Mendoza

**Affiliations:** 1 Biomedicine Laboratory, Research Center of Faculty of Health Sciences, Universidad Peruana de Ciencias Aplicadas, Lima 15087, Peru; pcmeebon@upc.edu.pe (E.B.-V.d.V.); miguel.aguilar@upc.pe (M.A.A.-L.); pcmeytar@upc.edu.pe (Y.T.-C.); deysi3aguilar98@gmail.com (D.A.-L.); pcmedden@upc.edu.pe (D.D.-H.); angela.cornejo@upc.edu.pe (A.C.-T.); ronald.aquino@upc.pe (R.A.-O.); 2 Oncology Unit, Hospital Regional Docente de Cajamarca, Cajamarca 06003, Peru; suspi_jg@hotmail.com; 3 Professional Academic School of Obstetrics, Universidad Nacional de Cajamarca, Cajamarca 06003, Peru; 4 Instituto de Investigacion de Enfermedades Infecciosas, Lima 15008, Peru

**Keywords:** human papillomavirus 16, papillomavirus infections, uterine cervical neoplasms, epitopes, T-lymphocyte, nanopore sequencing

## Abstract

Background: HPV16 is the most prevalent high-risk genotype associated with cervical cancer, yet the genetic variability and immune potential of the replication protein E1 are less characterized in asymptomatic infections. We assessed HPV16 E1 diversity and predicted conserved T-cell epitopes. Methods: We conducted a descriptive cross-sectional study of cervical samples from women undergoing HPV screening. HPV was detected with universal primers and then HPV16-specific PCR. Thirty HPV16-positive samples underwent full-length *E1* amplification and nanopore amplicon sequencing. Variability and phylogeny were analyzed with Clustal Omega and MEGA (maximum likelihood). MHC class I and II epitopes were predicted with the IEDB using HLA alleles representative of South American populations and evaluated for conservation, toxicity, allergenicity, and population coverage. Results: Mutations were detected in 14/30 samples, while 16 sequences matched the reference (GenBank: NC_001526.3). European lineages (A1–A3) predominated, with one sequence in the Asian-American lineage D. Seven highly conserved MHC I epitopes and 37 conserved MHC II epitopes were identified. Epitopes mapped to multiple regions across the *E1* sequence. Predicted global coverage was 94.38% for MHC I, 83.75% for MHC II, and 99.09% combined. Conclusions: HPV16 E1 is highly conserved and contains candidate T-cell targets with broad predicted coverage, supporting evaluation for future vaccine or immunotherapy strategies.

## 1. Introduction

Human papillomavirus (HPV) is currently the most common sexually transmitted infection worldwide, with a lifetime acquisition probability exceeding 80% among sexually active individuals [1]. Globally, an estimated 630 million people are currently infected with HPV, and genotypes 16 and 18 account for approximately 5% of all cancers worldwide [2,3]. The global incidence of cervical cancer exceeds 660,000 cases per year, with more than 300,000 deaths, disproportionately concentrated in low- and middle-income countries [4]. Peru is part of this challenging regional scenario and exhibits particular epidemiological characteristics. In 2022, 4809 cervical cancer cases were reported, leading to 2545 deaths and making it the second leading cause of female mortality in the country [5].

Human papillomavirus type 16 (HPV16) belongs to the genus Alphapapillomavirus and is the most prevalent oncogenic high-risk genotype worldwide, responsible for approximately 60% of cervical cancers and more than 90% of HPV-associated oropharyngeal carcinomas [6]. Its genome is a circular, double-stranded DNA molecule of approximately 7900 base pairs, with a complex and tightly regulated organization [7]. While most immunological and molecular studies have focused on the viral oncoproteins E6 and E7 due to their direct role in cellular transformation, other early proteins such as E1 have received comparatively limited attention despite their essential role in the viral life cycle. The E1 protein is the only HPV-encoded enzyme with intrinsic catalytic activity, functioning as an ATP-dependent DNA helicase required for viral replication. Beyond its canonical role, E1 is highly conserved across HPV16 variants due to strong functional constraints, making it a potentially stable target for immune recognition. Importantly, recent evidence suggests that non-oncogenic early proteins can also contribute to T-cell-mediated immune responses, highlighting their relevance as alternative targets for immunotherapeutic strategies. In this context, the relative conservation of E1 compared to the higher variability observed in E6 and E7 [8] may offer advantages for the identification of broadly recognized T-cell epitopes.

Massively parallel sequencing has revealed substantial intra-type variability, enabling HPV16 to be classified into phylogenetic variants with biologically meaningful differences. HPV16 genetic variability is not neutral; it has profound implications for viral pathogenesis and the risk of neoplastic progression [6,9,10]. Epidemiological studies have shown that women infected with variants from the B, C, and D lineages (non-European) have a significantly higher risk of progressing to high-grade cervical lesions and invasive cancer [6,11,12].

Clearance of HPV16 infection critically depends on the activation of an effective cellular immune response. The most relevant target antigens for this response are the early proteins, which harbor a higher density of epitopes recognized by cellular immunity. In silico prediction is particularly relevant for studies focused on specific populations. Because epitope presentation is restricted by HLA type and allele frequencies vary across populations, identifying promiscuous epitopes that are conserved among circulating regional variants is essential [13,14]. This enables the design of immune-monitoring strategies and potential vaccine candidates with optimized population coverage. In this study, we assessed genetic variability in the *E1* gene and identified HPV16 variants circulating in an asymptomatic population in our setting. Additionally, we examined how these variations may be relevant to immune responses and viral persistence.

## 2. Materials and Methods

### 2.1. Study Design and Ethical Considerations

We conducted an observational, cross-sectional, descriptive study aimed at characterizing genetic variability in the *E1* gene of HPV16 and evaluating its immunogenic potential through in silico prediction of T-cell epitopes. The study was performed in accordance with the ethical principles of the Declaration of Helsinki and was approved by the corresponding institutional ethics committee. All participants provided written informed consent prior to sample collection.

### 2.2. Study Population and Sample Collection

Cervical specimens were obtained from asymptomatic women attending routine gynecological check-ups at the Hospital Regional Docente de Cajamarca, Peru, during 2022–2024. Samples were collected by cervical brushing using sterile devices, subsequently preserved in an appropriate transport medium (PBS), and stored under controlled conditions until molecular processing. A total of 76 HPV16-positive samples were included in downstream analyses.

### 2.3. Genetic Material Extraction

Total DNA was extracted from cervical samples using a standardized nucleic acid extraction protocol according to the manufacturer’s instructions (CWBio Viral DNA/RNA Extraction; CWBIO, Taizhou, China). DNA integrity was assessed by agarose gel electrophoresis.

### 2.4. HPV16 Detection and Typing

Initial HPV detection was performed by conventional polymerase chain reaction (PCR) using universal HPV primers. HPV-positive samples were then subjected to a second PCR using HPV16-specific primers and previously described conditions [15] to confirm the presence of this oncogenic high-risk genotype. Amplicons were visualized by agarose gel electrophoresis and documented under blue light illumination.

### 2.5. HPV16 E1 Amplification and Sequencing

The full-length HPV16 *E1* gene was amplified by conventional PCR using specific primers (HPV16-E1F 5′-GGACAAGCAGAACCGGA-3′ and HPV16-E1R 5′-CGTCTTGTAATGTCCACTTTTC-3′), designed to cover the entire coding region [16]. PCR cycling conditions were 95 °C for 2 min; 50 cycles of 95 °C for 30 s, 55 °C for 45 s, and 72 °C for 2 min 30 s; followed by 72 °C for 10 min and a final hold at 10 °C. Positive PCR products were purified using the SpinPrep™ Gel DNA kit (Merck KGaA, Darmstadt, Germany) and stored at −80 °C until analysis. Amplicon sequencing of the complete E1 gene was performed using an Oxford Nanopore Technologies platform with the Rapid Barcoding Kit (SQK-RBK114), enabling long-read coverage across the full-length target region. Raw sequencing data were acquired using MinKNOW software, version 25.05.14, followed by basecalling and demultiplexing using Dorado (v1.3) under high-accuracy settings. Quality control filtering was applied to remove reads with a Phred quality score below Q10 prior to downstream analyses. Consensus sequence generation was conducted using the EPI2ME workflow wf-amplicons (v1.1.4). Reads were aligned through reference-guided assembly against the HPV16 reference genome (GenBank: NC_001526.3). To ensure reliable variant detection and minimize sequencing- and PCR-associated errors inherent to amplicon-based nanopore sequencing, a stringent minimum coverage threshold of 100× was applied, exceeding the recommended minimum coverage of 60× for variant calling [17].

### 2.6. Genetic Variation and Phylogenetic Analysis

Consensus *E1* sequences were aligned using the Clustal Omega algorithm version 1.2.4. Genetic variability, including nucleotide substitutions and inferred amino acid changes, was assessed in MEGA against the reference genome (GenBank: NC_001526.3). Phylogenetic analysis was performed in MEGA based on multiple sequence alignments generated with Clustal Omega. Trees were inferred using the neighbor-joining method, applying appropriate evolutionary models selected by statistical criteria, and clade robustness was assessed by bootstrap analysis with 1000 replicates. Study sequences were compared with representative HPV16 lineage variants retrieved from GenBank: HQ644283.1(A1), HQ644268.1(A1), HQ644280.1(A1), HQ644282.1(A1), AF536179.1(A2), HQ644236.1(A3), HQ644248.1(A4), HQ644251.1(A4), HQ644235.1(A4), HQ644240.1(B1), HQ644238.1(B1), HQ644290.1(B1), HQ644298.1(B2), HQ644237.1(C1), HQ644239.1(C), HQ644249.1(C), HQ644250.1(C), AF472509.1(C), HQ644257.1(D1), HQ644279.1(D2), HQ644281.1(D2), HQ644263.1(D2), HQ644277.1(D2), HQ644247.1(D3), HQ644253.1(D3), HQ644255.1(D3), AF402678.1(D3), NC001526.3(A1), AF536180.1(B1), KU053910.1(B2), KU053914.1(B4), HQ644244.1(C2), KU053921.1(C3), KU053922.1(C4), AY686579.1(D2), KU053933.1(D4), and AF534061.1(A4).

### 2.7. In Silico Prediction of T-Cell Epitopes

T-cell epitope prediction from the E1 protein was performed using bioinformatic tools available through the Immune Epitope Database (IEDB). The HLA class I and II alleles were selected based on recommendations from the IEDB, prioritizing those with the highest reported frequencies in South American populations according to the Net Allele Frequency Database, as described by Requena et al. The complete list of alleles included in this study is provided in Supplementary Table S2. Candidate epitopes were evaluated for peptide–HLA binding affinity (NetMHCpan v4.0 and NetMHCIIpan v4.1) [18], allergenicity potential (pLM4Alg) [19], predicted toxicity (CSM-Toxin) [20], and estimated population coverage (IEDB Population Coverage Tool). To assess epitope conservation, a dataset comprising 495 HPV16 E1 protein sequences was retrieved from the UniProt databases. The query was performed in February 2026. Only complete *E1* sequences annotated as HPV16 were included. Sequences with truncations, ambiguous residues, or incomplete annotations were excluded. Redundant entries were removed to avoid duplication bias. Epitope conservation analysis was performed on these sequences using the IEDB Epitope Conservancy Analysis tool. Finally, the IEDB was searched using HPV16 as the source organism and the E1 protein as the antigen. The search was restricted to epitopes with positive assays for T-cell responses or MHC binding. Epitope identification was performed by exact matching of peptide sequences to retrieve previously validated sequences. This database search was conducted in February 2026.

## 3. Results

### 3.1. Genetic Variation Analysis of the HPV16 E1 Gene

**Figure 1:**
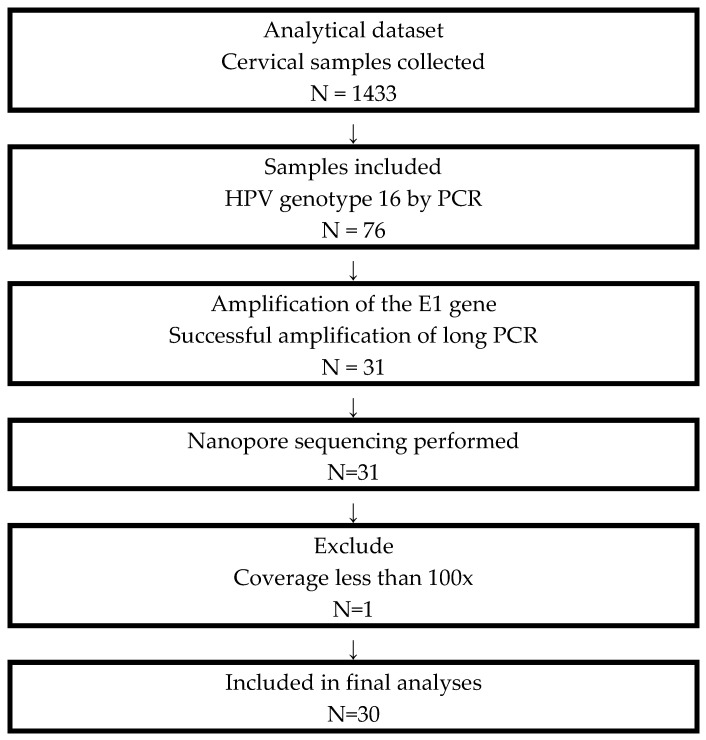
Sample selection and processing flow.

Although 76 samples were confirmed as positive for HPV16, only sequences meeting predefined quality criteria were included in subsequent analyses (Figure 1). Samples had to exhibit successful amplification of the entire E1 gene, sufficient DNA for library preparation, and sequencing reads achieving a minimum coverage threshold of 100× in more than 95% of the target region. After quality filtering, 30 high-confidence consensus sequences were obtained for genetic variability and phylogenetic analyses.

To assess potential selection bias, the basic epidemiological characteristics of the sequenced samples (n = 30) were compared with those of the total HPV16-positive cohort (n = 76). No substantial statistical differences were observed in age distribution (18.4 ± 2.5), sampling period, or clinical characteristics, suggesting that the sequenced subset is representative of the overall HPV16-positive population.

Subsequently, comparison of the obtained sequences with the reference sequence (GenBank: NC_001526.3) revealed nucleotide variation in 14 isolates (46.7%), while 16 sequences (53.3%) were identical to the prototype strain. Analysis of the complete HPV16 E1 coding region identified 71 nucleotide variants distributed across the entire gene. Most variants corresponded to nonsynonymous (n = 34) and synonymous (n = 37) substitutions dispersed throughout the coding sequence (Table 1). Among the detected variants, G1515A was the most frequent, observed in 20.0% of samples (n = 6), followed by G1103A and G1163A, each present in 6.7% of isolates (n = 2). All remaining variants occurred at an individual frequency of 3.3%, corresponding to changes detected in a single isolate.

At the amino acid level, multiple nonsynonymous substitutions were identified across functional domains of the E1 protein. Within the disordered N-terminal region, the A5T substitution (G877A) was observed, and additional changes such as D41G, D47Y, and D55H mapped to early regions of the protein. The Q78E variant and the conservative R80K substitution were detected at positions proximal to regions implicated in early regulatory functions. In the domain associated with nuclear export and nuclear localization signals, the G100E and G100V variants were identified, suggesting changes in regions potentially involved in intracellular trafficking of the viral protein. In the DNA-binding region, the C168S substitution was observed, while other amino acid changes such as Q188E and L198R were distributed across intermediate segments of the polypeptide. The helicase domain—functionally critical for viral replication—harbored multiple nonsynonymous substitutions (Table 1), indicating genetic diversity even within structurally conserved regions of E1. Finally, a single substitution, S618Y, was detected in the disordered C-terminal region (Table 1).

### 3.2. Phylogenetic Analysis of HPV16

**Figure 2:**
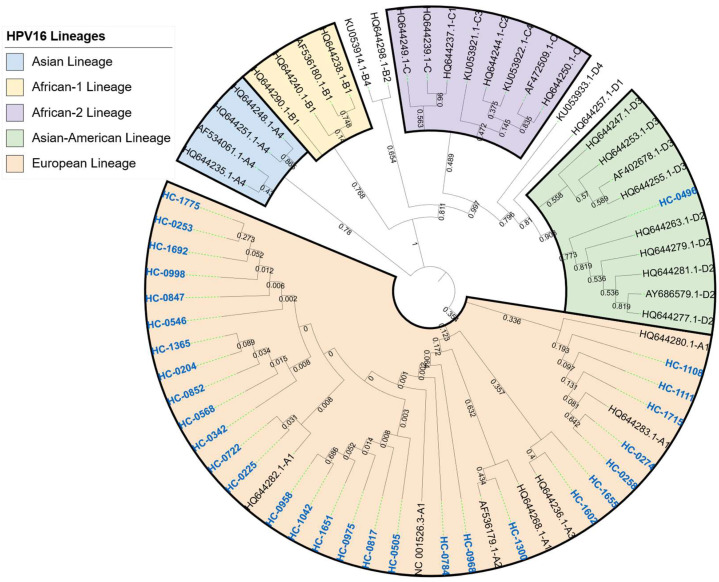
A phylogenetic tree based on full-length HPV16 E1 gene sequences. The obtained sequences are shown in bold blue. The analyzed sequences clustered predominantly within the European lineage (A1–A3), with a single sample belonging to the Asian-American lineage D. Representative reference sequences from lineages A, B, C, and D were included for phylogenetic classification.

Phylogenetic analysis of the 30 HPV16 E1 gene sequences showed a clear predominance of European lineage variants. Overall, 29 isolates clustered within sublineages A1, A2, and A3, whereas only one sample was classified within lineage D (Asian-American). No sequences corresponding to lineage B (African-1), lineage C (African-2), or sublineage A4 (Asian) were identified among the analyzed samples. The phylogenetic distribution of the sequences is shown in Figure 2.

### 3.3. Assessment of the Impact of Variants on T-Cell Epitope Recognition

**Figure 3:**
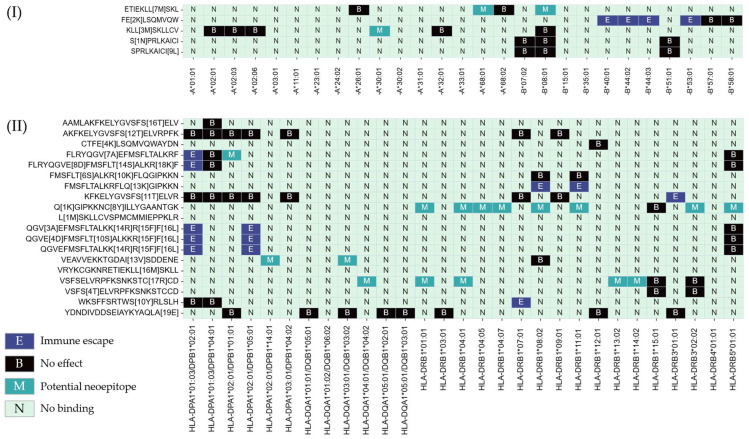
The predicted impact of E1 mutations on T-cell epitope binding to HLA class I and class II molecules. The figure illustrates changes in binding profiles between native and mutated E1-derived epitopes across a panel of representative HLA class I (**top**) and class II (**bottom**) alleles. Cells labeled E indicate loss of binding of native epitopes due to sequence mutations (shown in brackets), suggesting potential immune escape. B indicates conserved binding for both native and mutated epitopes, M denotes predicted binding exclusively for mutated epitopes (possible neoepitope generation), and N denotes no binding.

To evaluate the potential functional impact of the identified mutations on immune recognition, we analyzed the HLA class I and class II binding capacity of E1-derived epitopes. Peptide–HLA binding is an essential step for antigen presentation by antigen-presenting cells and the subsequent activation of T lymphocytes, as well as for the induction of T-cell-dependent humoral responses. For this analysis, epitopes containing mutations were assessed alongside their native homologous sequences using HLA-binding prediction tools. Predicted interactions with representative HLA alleles were compared to identify potential losses or gains in immune recognition. Prediction results for MHC class II (CD4^+^) and MHC class I (CD8^+^) molecules are shown in Figure 3. The analysis indicated that eight previously reported CD4^+^ T-cell epitopes were affected by mutations present in the analyzed variants, suggesting a potential loss of immune recognition in specific HLA contexts. In contrast, only one CD8^+^-restricted epitope showed reduced binding associated with amino-acid variation. In addition, some mutations generated new HLA-binding profiles. Overall, four mutated epitopes exhibited a gain of predicted binding to MHC class II molecules, whereas two epitopes showed new predicted binding to MHC class I, suggesting the possible emergence of neoepitopes driven by genetic variability in the E1 gene (Figure 3).

### 3.4. In Silico Identification of Conserved T-Cell Epitopes and Assessment of Their Characteristics

**Figure 4:**
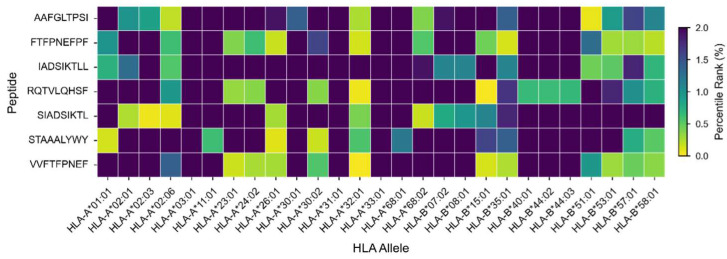
The predicted binding affinity of conserved MHC class I epitopes to representative HLA class I alleles. Heatmap illustrating the predicted interactions between conserved CD8^+^ T-cell epitopes and the analyzed HLA-I alleles. Color intensity represents relative binding affinity scores, where a lower percentile rank indicates stronger binding to the corresponding MHC.

**Figure 5:**
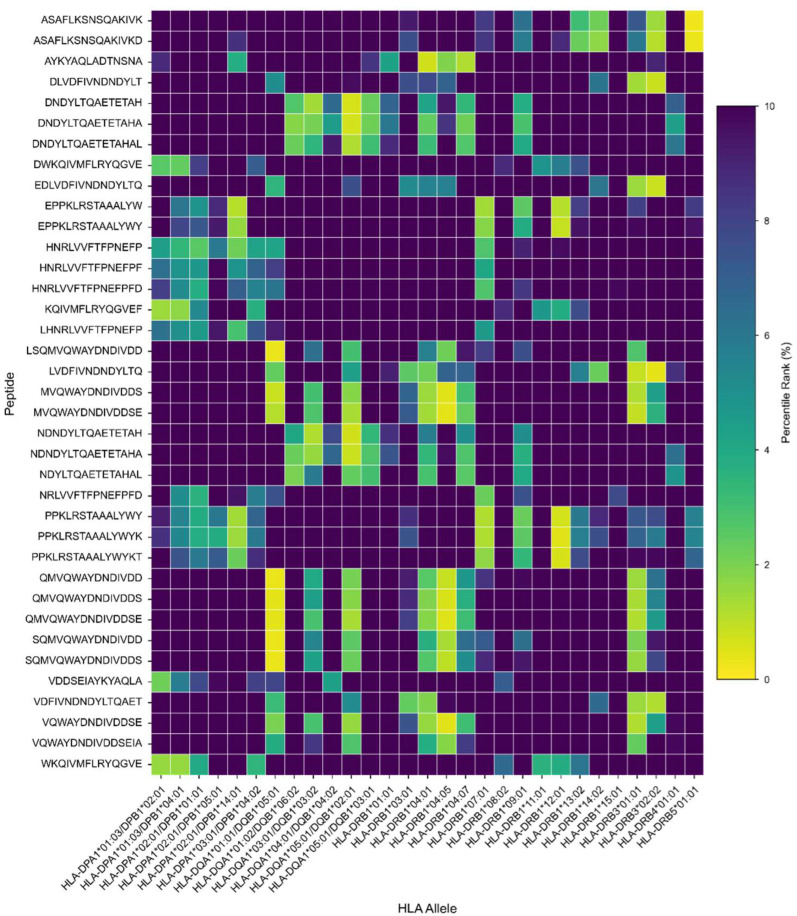
The predicted binding affinity of conserved MHC class II epitopes with representative HLA class II alleles. Heat map showing the predicted interactions between conserved CD4^+^ T-cell epitopes and selected HLA-II alleles in diverse populations. Color intensity represents relative binding affinity comparisons, where a lower percentile rank reflects better binding to their MHC.

**Figure 6:**
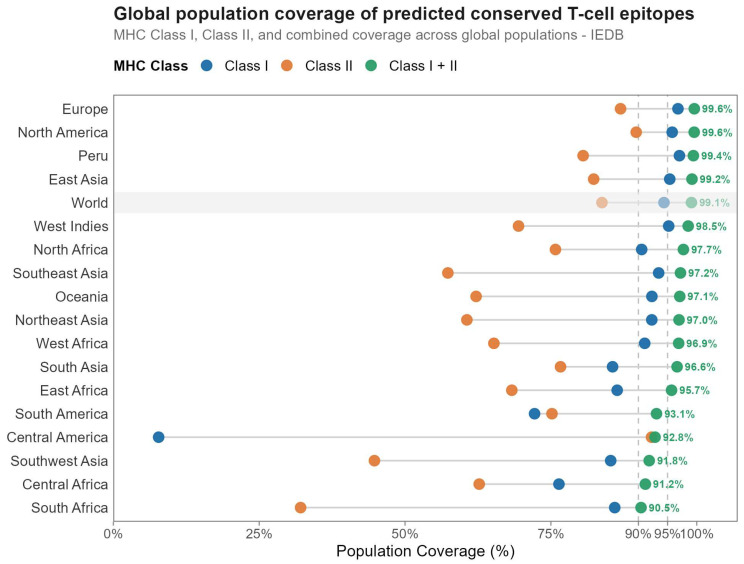
Global population coverage of predicted conserved T-cell epitopes derived from the HPV16 E1 protein. Estimated population coverage for MHC class I, MHC class II, and the combined response, calculated using the IEDB Population Coverage tool.

To identify immunologically relevant regions with potential utility for rational immunogen design, we performed in silico prediction of highly conserved T-cell epitopes across the analyzed E1 sequences. We prioritized epitopes displaying high conservation, adequate major histocompatibility complex (MHC) binding affinity, and favorable immunological safety profiles. In total, 44 conserved epitopes were identified, comprising 7 MHC class I-restricted (CD8^+^) epitopes and 37 MHC class II–restricted (CD4^+^) epitopes. Selected epitopes showed high conservation, with sequence coverage ranging from 80% to 98.18% and a minimum identity of 77.78%, indicating low susceptibility to the genetic variability observed among circulating variants (Table 2). Immunological safety assessment indicated that all predicted epitopes were classified as non-toxic and non-allergenic, supporting their potential applicability. In addition, at the time of the analysis, no exact matches were identified for the predicted epitopes in the IEDB with our search strategy. Structural mapping revealed a differential distribution of epitopes across key functional domains of the E1 protein: 10 epitopes in the disordered N-terminal region associated with regulatory functions, 10 epitopes in the DNA-binding domain (5 MHC I and 5 MHC II), and 24 epitopes within the helicase domain, including 2 MHC I and 22 MHC II. The full list of associated alleles is provided in Table 2 and the MHC alleles that bind to these are detailed in Supplementary Table S1. HLA binding profiles demonstrated broad recognition potential across multiple alleles for both MHC class I and class II. Heatmaps showed robust binding affinity patterns between conserved epitopes and representative HLA alleles across diverse human populations, indicating high antigen-presentation potential (Figure 4 and Figure 5). Population coverage analysis revealed high predicted global immune recognition for the selected epitopes, with estimated coverage values of 94.38% for MHC class I, 83.75% for MHC class II, and 99.09% for the combined response (Figure 6). At the regional level, slightly higher combined coverage was observed for Peru (99.4%), consistent with the prioritization of HLA alleles enriched in this population. In contrast, a moderate decrease in coverage was observed at the broader South American level (93.1%). Greater variability in predicted coverage was observed across other geographic regions, including Central America, Southeast Asia, and Central and Southern Africa, where lower combined coverage values were estimated. These results highlight differences in HLA allele distribution across populations and their impact on predicted epitope recognition.

## 4. Discussion

This study characterized HPV16 E1 genetic variability in cervical samples from asymptomatic women and assessed its potential impact on antigenicity through in silico prediction of conserved T-cell epitopes.

Using samples from asymptomatic women allows for a better understanding of HPV16 infection in its early stages, before the development of clinically detectable lesions. This approach enables the characterization of viral genetic variability and epitope profiles in a context less influenced by host immune selection, which could favor the emergence of escape variants, potentially altering the epitope repertoire. Therefore, the patterns observed in this study could reflect early interactions between the virus and the host, and future studies comparing different clinical stages would be valuable for further elucidating the role of E1 variability in disease progression [8].

Although HPV16 lineage classification is traditionally based on whole-genome sequences, previous studies have demonstrated that individual coding regions, including *E1*, retain sufficient phylogenetic signal to reliably resolve major HPV16 lineages. This is largely attributable to the low recombination rate and strong genome-wide linkage characteristic of papillomaviruses [21]. However, we acknowledge that lineage assignment based on a single genomic region may limit the resolution of closely related sublineages compared to whole-genome approaches, which remain the reference standard. This represents a limitation of the present study and highlights the value of future analyses integrating whole-genome sequencing data.

Our findings indicate a low overall frequency of nucleotide variation, a predominance of the European lineage (A1–A3), and the presence of multiple highly conserved T-cell epitopes with broad predicted population coverage, suggesting that E1 may represent a relatively stable immunological target despite viral genetic diversity. The predominance of European lineage variants observed here is consistent with previous reports describing the widespread global distribution of A variants [22,23,24], particularly in cervical infections from Latin American populations [11,25,26] and Peruvian population [27] which coincides with our findings. Although multiple nucleotide substitutions were detected across E1, most occurred at low frequency and in single samples, indicating limited genetic diversification. The identification of nonsynonymous substitutions within the helicase domain is particularly relevant given that this region harbors highly conserved motifs essential for ATP-dependent DNA unwinding and viral replication. These motifs are generally under strong purifying selection, suggesting that most observed substitutions are likely compatible with preserved enzymatic function. However, even subtle amino acid changes may affect protein folding, stability, or interactions with host replication machinery, potentially influencing viral fitness [9]. Extensive alterations in these regions could compromise viral replication, thereby evolutionarily limiting the accumulation of functionally disruptive mutations [28]. Moreover, conserved C-terminal domains of HPV11 E1 have been shown to be required for oligomerization and efficient viral replication, reinforcing the notion that the conservation observed reflects strict functional constraints [29]. In contrast, the N-terminal region of E1 is less conserved across viral types and contains variable nuclear localization and export signals, which may facilitate host-specific adaptations without affecting the essential helicase activity [30].

The in silico assessment of epitope loss and neoepitope generation suggests that specific nucleotide variations in HPV16 E1 may modulate T-cell-mediated immune recognition by altering MHC-binding affinity. We observed that some mutations were associated with loss of previously recognized epitopes, whereas others generated new peptides with predicted binding to HLA class I and II molecules. This phenomenon has been described as a potential mechanism of viral immune evasion, whereby the accumulation of mutations within antigenic regions may reduce antigen presentation and alter activation of antigen-specific T cells [31]. However, given the dual nature of this process, a dynamic balance between viral adaptation and immune surveillance may exist, and the underlying mechanisms over time remain incompletely elucidated.

Historically, HPV immunology has focused on the E6 and E7 oncoproteins; nevertheless, emerging evidence indicates that non-oncogenic early proteins, including E1, can also elicit robust T-cell responses. Immunological analyses in HPV16-associated cancers have identified significant cytotoxic responses directed against E1- and E2-derived epitopes shared across patients, indicating that these proteins constitute relevant antigen sources recognized by T lymphocytes [32]. In addition, CD8^+^ responses against E1 antigens have been shown to exhibit cross-reactivity across HPV genotypes, supporting the presence of conserved epitopes capable of inducing broad immunity [33]. These findings support the hypothesis that conserved replicative proteins may represent more stable immune targets than proteins under stronger selective pressure. In this context, the identification of highly conserved epitopes with high predicted population coverage is one of the most relevant findings of the present study. Current prophylactic HPV vaccines primarily induce humoral immunity against capsid proteins but have limited effectiveness against established infections [34]. By contrast, T-epitope-based strategies aim to activate cellular immunity capable of eliminating infected cells through recognition of conserved viral peptides. Targeting conserved genomic regions has been proposed to reduce the likelihood of immune escape and increase applicability across viral variants [35]. The high structural conservation of the helicase domain observed in our results, together with the broad predicted HLA coverage, suggests that E1 may be a promising candidate for the development of multiepitope therapeutic vaccines or immunomodulatory strategies targeting persistent HPV16 infection. The predominance of European lineage variants (A1-A3) in our dataset is consistent with previous reports in Latin American populations. However, given that non-European lineages (B, C, and D) have been associated with a higher risk of malignant progression, the limited representation of these variants in our study could affect the generalizability of our findings. To mitigate this limitation, we integrated an additional 495 global sequences into our conservation analysis, providing a broader context for the robustness of the predicted T-cell epitopes against known worldwide variability. Therefore, the epitope profiles and conservation patterns described here may primarily reflect the immunogenetic landscape of European HPV16 variants. Future studies incorporating a broader representation of non-European lineages will be essential to determine whether similar patterns hold for variants with different oncogenic potential.

Identifying T-cell epitopes using computational approaches represents a valuable first step in characterizing viral immunogenic regions, especially when integrated with sequence data from circulating variants. By combining genomic sequencing with immunoinformatics, this study allows us to assess how naturally occurring mutations can influence antigen presentation and population immunity. While computational predictions do not replace experimental validation, they are widely used to prioritize candidate epitopes for further functional studies and vaccine design. In this context, our findings help refine the set of potential immunogenic targets within the E1 protein and highlight regions that warrant further investigation. Ultimately, however, in vitro functional studies and immunological analyses in clinical cohorts will be required to experimentally validate the immunogenicity and biological relevance of the proposed epitopes.

## 5. Conclusions

Analysis of HPV16 E1 genetic variability in cervical samples from asymptomatic women demonstrated high genomic conservation, with a predominance of the European lineage (A1–A3) and low phylogenetic diversity. The in silico analyses indicated that certain mutations may alter binding to HLA molecules, leading either to loss of previously recognized epitopes or to the emergence of new candidate epitopes, suggesting potential mechanisms of viral immune modulation. Furthermore, the high estimated population coverage for conserved epitopes supports the potential of E1 as a source of antigenic candidates for future immunotherapy strategies or rational design of therapeutic HPV16 vaccines. Overall, these results expand current knowledge of HPV16 genetic diversity and its immunological landscape in subclinical infections, providing a molecular basis for future research aimed at understanding virus–host interactions and developing targeted immunological interventions.

## Figures and Tables

**Table 1:** Variations identified in the HPV16 E1 gene (European sublineage A1, GenBank: NC_001526.3) and their potential impact on amino acid substitutions in the E1 protein.

Variation	Frequency (%)	Number of Variant Samples	Substitution	Domain in the E1 Protein
G877A	3.3	1	A5T	Disordered N-Terminal
G913T	3.3	1	-	
G954A	3.3	1	-	
A961G	3.3	1	-	
A986G	3.3	1	D41G	
A996G	3.3	1	-	
G1003T	3.3	1	D47Y	
T1006C	3.3	1	-	
G1027C	3.3	1	D55H	
G1033A	3.3	1	-	
A1041G	3.3	1	-	
G1069A	3.3	1	-	
C1096G	3.3	1	Q78E	
G1103A	6.7	2	R80K	
C1120T	3.3	1	-	
A1122G	3.3	1	-	
A1133G	3.3	1	-	
G1163A	6.7	2	G100E	Nuclear export and localization signal
G1163T	3.3	1	G100V	
G1184A	3.3	1	S107N	
T1200C	3.3	1	-	
T1200G	3.3	1	-	
A1207C	3.3	1	-	
T1244C	3.3	1	-	
A1345C	3.3	1	-	
T1366A	3.3	1	C168S	DNA-binding region
C1377T	3.3	1	-	
C1426G	3.3	1	Q188E	
T1457G	3.3	1	L198R	
T1486C	3.3	1	-	
G1515A	20.0	6	-	
T1522A	6.7	2	S220T	
T1561C	3.3	1	C233R	
C1624T	3.3	1	-	
A1668G	3.3	1	-	
A1704G	3.3	1	-	
C1744A	3.3	1	L294M	
A1842G	3.3	1	I326M	
C1885G	3.3	1	Q341E	
T1901C	3.3	1	L346S	Helicase domain
G1930A	3.3	1	E356K	
C2012A	3.3	1	A383E	
A2027G	3.3	1	N388S	
C2041T	3.3	1	-	
C2054T	3.3	1	S397L	
G2150T	3.3	1	R429I	
T2216C	3.3	1	V451A	
G2220C	3.3	1	E452D	
C2237G	3.3	1	T458S	
G2249A	3.3	1	R462K	
T2254C	3.3	1	-	
C2257A	3.3	1	Q465K	
C2262T	3.3	1	-	
G2279A	3.3	1	C472Y	
C2287T	3.3	1	-	
T2343C	3.3	1	-	
C2344T	3.3	1	-	
T2376C	3.3	1	-	
C2405T	3.3	1	A514V	
A2475C	3.3	1	R537S	
T2499C	3.3	1	-	
A2529T	3.3	1	-	
T2586C	3.3	1	-	
T2595G	3.3	1	-	
T2595C	3.3	1	-	
A2608C	3.3	1	-	
T2631A	3.3	1	-	
T2644C	3.3	1	F594L	
G2650A	3.3	1	E596K	
T2679C	3.3	1	-	
C2717A	3.3	1	S618Y	Disordered C-Terminal

**Table 2:** Evaluation and characterization of T-cell epitopes with high immunological potential.

Epitope	Number of Hits/MHC Allele	100% Identity (%)	Minimum Identity (%)	E1 Domain	Length (aa)	Molecular Weight (Da)	Nonpolar Amino Acids (n)	Polar Amino Acids (n)	Charged Amino Acids (n)	Net Charge	Hydrophobicity
AAFGLTPSI	6/HLA-A 4/HLA-B	97.78% (484/495)	88.89%	DNA-BD	9	876.02	7	2	0	0	1.24
SIADSIKTL	7/HLA-A 3/HLA-B	98.18% (486/495)	77.78%	DNA-BD	9	947.1	4	5	2	0	0.54
VVFTFPNEF	7/HLA-A 5/HLA-B	97.58% (483/495)	77.78%	HD	9	1099.25	6	3	1	−1	0.83
FTFPNEFPF	7/HLA-A 5/HLA-B	98.18% (486/495)	77.78%	HD	9	1145.28	6	3	1	−1	0.03
RQTVLQHSF	5/HLA-A 3/HLA-B	94.95% (470/495)	77.78%	DNA-BD	9	1115.26	3	6	2	1.09	−0.6
STAAALYWY	5/HLA-A 3/HLA-B	96.16% (476/495)	77.78%	DNA-BD	9	1045.16	7	2	0	0	0.47
IADSIKTLL	4/HLA-A 4/HLA-B	98.18% (486/495)	88.89%	DNA-BD	9	973.18	5	4	2	0	1.06
HNRLVVFTFPNEFP	1/HLA-DQ 3/HLA-DR 6/HLA-DP	96.57% (478/495)	78.57%	HD	14	1716.96	8	6	3	0.09	−0.11
VQWAYDNDIVDDSE	3/HLA-DQ 7/HLA-DR	96.77% (479/495)	85.71%	HD	14	1668.69	6	8	5	−5	−0.91
DNDYLTQAETETAH	5/HLA-DQ 6/HLA-DR	89.49% (443/495)	85.71%	N-Ter	14	1607.61	4	10	5	−3.91	−1.44
EPPKLRSTAAALYW	6/HLA-DR 5/HLA-DP	95.15% (471/495)	85.71%	DNA-BD	14	1602.85	9	5	3	1	−0.41
QMVQWAYDNDIVDD	3/HLA-DQ 8/HLA-DR	97.37% (482/495)	92.86%	HD	14	1711.82	7	7	4	−4	−0.72
LVDFIVNDNDYLTQ	2/HLA-DQ 10/HLA-DR	94.55% (468/495)	92.86%	N-Ter	14	1668.82	7	7	3	−3	0.02
PPKLRSTAAALYWY	9/HLA-DR 6/HLA-DP	94.95% (470/495)	85.71%	DNA-BD	14	1636.91	10	4	2	2	−0.26
KQIVMFLRYQGVEF	4/HLA-DR 4/HLA-DP	80.00% (396/495)	85.71%	HD	14	1758.11	9	5	3	1	0.26
DLVDFIVNDNDYLT	2/HLA-DQ 6/HLA-DR	94.34% (467/495)	85.71%	N-Ter	14	1655.78	7	7	4	−4	0.02
AYKYAQLADTNSNA	1/HLA-DQ 5/HLA-DR 2/HLA-DP	95.76% (474/495)	85.71%	HD	14	1529.63	7	7	2	0	−0.79
VDDSEIAYKYAQLA	2/HLA-DQ 1/HLA-DR 5/HLA-DP	97.17% (481/495)	78.57%	HD	14	1585.73	8	6	4	−2	−0.24
WKQIVMFLRYQGVE	4/HLA-DR 5/HLA-DP	80.40% (398/495)	85.71%	HD	14	1797.15	9	5	3	1	−0.01
MVQWAYDNDIVDDS	3/HLA-DQ 6/HLA-DR	96.97% (480/495)	92.86%	HD	14	1670.77	7	7	4	−4	−0.53
EPPKLRSTAAALYWY	6/HLA-DR 4/HLA-DP	94.95% (470/495)	86.67%	DNA-BD	15	1766.03	10	5	3	1	−0.47
SQMVQWAYDNDIVDD	3/HLA-DQ 7/HLA-DR	97.37% (482/495)	93.33%	HD	15	1798.9	7	8	4	−4	−0.73
DNDYLTQAETETAHA	5/HLA-DQ 6/HLA-DR	89.29% (442/495)	86.67%	N-Ter	15	1678.69	5	10	5	−3.91	−1.23
PPKLRSTAAALYWYK	9/HLA-DR 6/HLA-DP	94.75% (469/495)	80.00%	DNA-BD	15	1765.09	10	5	3	3	−0.5
DWKQIVMFLRYQGVE	4/HLA-DR 4/HLA-DP	80.40% (398/495)	86.67%	HD	15	1912.24	9	6	4	0	−0.24
NDYLTQAETETAHAL	4/HLA-DQ 4/HLA-DR	90.30% (447/495)	86.67%	N-Ter	15	1676.76	6	9	4	−2.91	−0.74
HNRLVVFTFPNEFPF	1/HLA-DQ 1/HLA-DR 6/HLA-DP	96.36% (477/495)	80.00%	HD	15	1864.14	9	6	3	0.09	0.09
LHNRLVVFTFPNEFP	1/HLA-DQ 1/HLA-DR 6/HLA-DP	96.57% (478/495)	80.00%	HD	15	1830.12	9	6	3	0.09	0.15
ASAFLKSNSQAKIVK	8/HLA-DR	96.97% (480/495)	80.00%	HD	15	1591.87	7	8	3	3	−0.03
NDNDYLTQAETETAH	5/HLA-DQ 4/HLA-DR	89.09% (441/495)	86.67%	N-Ter	15	1721.71	4	11	5	−3.91	−1.58
NRLVVFTFPNEFPFD	2/HLA-DQ 3/HLA-DR 4/HLA-DP	96.36% (477/495)	86.67%	HD	15	1842.08	9	6	3	−1	0.07
QMVQWAYDNDIVDDS	3/HLA-DQ 6/HLA-DR	96.57% (478/495)	93.33%	HD	15	1798.9	7	8	4	−4	−0.73
MVQWAYDNDIVDDSE	3/HLA-DQ 6/HLA-DR	96.77% (479/495)	86.67%	HD	15	1799.88	7	8	5	−5	−0.73
DNDYLTQAETETAHAL	5/HLA-DQ 5/HLA-DR	89.29% (442/495)	87.50%	N-Ter	16	1791.85	6	10	5	−3.91	−0.91
HNRLVVFTFPNEFPFD	1/HLA-DQ 3/HLA-DR 6/HLA-DP	96.16% (476/495)	81.25%	HD	16	1979.22	9	7	4	−0.91	−0.14
SQMVQWAYDNDIVDDS	3/HLA-DQ 7/HLA-DR	96.57% (478/495)	93.75%	HD	16	1885.98	7	9	4	−4	−0.73
ASAFLKSNSQAKIVKD	9/HLA-DR 1/HLA-DP	96.57% (478/495)	81.25%	HD	16	1706.96	7	9	4	2	−0.24
NDNDYLTQAETETAHA	5/HLA-DQ 6/HLA-DR	89.09% (441/495)	87.50%	N-Ter	16	1792.79	5	11	5	−3.91	−1.37
PPKLRSTAAALYWYKT	7/HLA-DR 5/HLA-DP	94.75% (469/495)	81.25%	DNA-BD	16	1866.19	10	6	3	3	−0.51
EDLVDFIVNDNDYLTQ	2/HLA-DQ 6/HLA-DR	93.94% (465/495)	87.50%	N-Ter	16	1913.03	7	9	5	−5	−0.42
VQWAYDNDIVDDSEIA	3/HLA-DQ 5/HLA-DR	96.36% (477/495)	87.50%	HD	16	1852.93	8	8	5	−5	−0.41
VDFIVNDNDYLTQAET	2/HLA-DQ 7/HLA-DR	89.90% (445/495)	93.75%	N-Ter	16	1856.96	7	9	4	−4	−0.37
QMVQWAYDNDIVDDSE	3/HLA-DQ 6/HLA-DR	96.36% (477/495)	87.50%	HD	16	1928.01	7	9	5	−5	−0.9
LSQMVQWAYDNDIVDD	3/HLA-DQ 6/HLA-DR	97.17% (481/495)	93.75%	HD	16	1912.06	8	8	4	−4	−0.44

HD: helicase domain; N-Ter: Disordered N-Terminal; DNA-BD: DNA binding region.

## Data Availability

The data presented in the study are openly available at https://doi.org/10.6084/m9.figshare.30267019.

## Supplementary Materials

The following supporting information can be downloaded at: https://www.mdpi.com/article/10.3390/pathogens15050526/s1, Table S1: MHC alleles. Table S2. Complete list of alleles included in this study.
